# Unravelling strong electronic interlayer and intralayer correlations in a transition metal dichalcogenide

**DOI:** 10.1038/s41467-021-27182-y

**Published:** 2021-11-30

**Authors:** T. J. Whitcher, Angga Dito Fauzi, D. Caozheng, X. Chi, A. Syahroni, T. C. Asmara, M. B. H. Breese, A. H. Castro Neto, A. T. S. Wee, M. Aziz Majidi, A. Rusydi

**Affiliations:** 1 Advanced Research Initiative for Correlated-Electron Systems (ARiCES), Department of Physics, National University of Singapore, 2 Science Drive 3, Singapore, 117576 Singapore; 2 Singapore Synchrotron Light Source, National University of Singapore, 5 Research Link, Singapore, 117603 Singapore; 3 Centre for Advanced 2D Materials, National University of Singapore, 2 Science Drive 3, Singapore, 117546 Singapore; 4 Department of Physics, University of Indonesia, Depok, 16424 Indonesia; 5 NUSSNI-NanoCore, National University of Singapore, Singapore, 117576 Singapore; 6 NUS Graduate School for Integrative Sciences and Engineering, Singapore, 117456 Singapore

**Keywords:** Phase transitions and critical phenomena, Electronic properties and materials

## Abstract

Electronic correlations play important roles in driving exotic phenomena in condensed matter physics. They determine low-energy properties through high-energy bands well-beyond optics. Great effort has been made to understand low-energy excitations such as low-energy excitons in transition metal dichalcogenides (TMDCs), however their high-energy bands and interlayer correlation remain mysteries. Herewith, by measuring temperature- and polarization-dependent complex dielectric and loss functions of bulk molybdenum disulphide from near-infrared to soft X-ray, supported with theoretical calculations, we discover unconventional soft X-ray correlated-plasmons with low-loss, and electronic transitions that reduce dimensionality and increase correlations, accompanied with significantly modified low-energy excitons. At room temperature, interlayer electronic correlations, together with the intralayer correlations in the *c*-axis, are surprisingly strong, yielding a three-dimensional-like system. Upon cooling, wide-range spectral-weight transfer occurs across a few tens of eV and in-plane *p–d* hybridizations become enhanced, revealing strong Coulomb correlations and electronic anisotropy, yielding a two-dimensional-*like* system. Our result shows the importance of strong electronic, interlayer and intralayer correlations in determining electronic structure and opens up applications of utilizing TMDCs on plasmonic nanolithrography.

## Abstract (web summary)

Electronic and interlayer correlations are expected to affect the electronic and optical properties of transition metal dichalcogenides. Here, by using spectroscopic ellipsometry in the broad energy range, the authors uncover new electronic transitions and correlated plasmons in bulk MoS_2_.

## Introduction

Electronic correlations have played an important role in generating many rich and exotic phase diagrams in strongly correlated electron systems^1–3^. These include colossal magnetoresistance (CMR)^4,5^, metal–insulator transitions^6,7^, and high-energy resonant excitons^8,9^, that are manifested through many-body interactions. Recently, strong electronic correlation effects have also been found to drive plasmons that are found in topological insulators such as bismuth selenide (Bi_2_Se_3_) when they are cooled^10,11^. This is due to the suppression of the electronic screening, which enhances long-range and high-energy electron–electron correlations, revealing topological insulators as strongly-correlated materials.

An important implication of electronic correlations is that high-energy bands, which are well beyond the optical regime, are tightly connected with low-energy bands, with the result being that low-energy properties, such as low-energy excitons, transports, or magnetism, may be determined by high-energy bands. These strong correlations can directly be probed. They show a unique spectral weight transfer, as a function of temperature or doping, which is a fingerprint of the complex dielectric function and loss function of a material, and involve high-energy and low-energy bands simultaneously fulfilling the charge conservation and *f*-sum rule^1,12–15^.

A group-VI low-dimensional transition metal dichalcogenide (TMDC) such as molybdenum disulphide (MoS_2_) is of particular interest because of its rich fundamental properties and potential applications. Recently, low-energy electronic properties of monolayer MoS_2_, have been heavily investigated due to its transformation from an indirect band-gap semiconducting material in its bulk form to a direct band-gap semiconductor^16–18^. Most studies have been focused on the formation of low-energy excitons and the effects of low-dimensional confinement^19–21^, which were shown to give rise to photoluminescence in the monolayers^22,23^, and the development of valleytronics based on the spin–orbit splitting of the valence and conduction bands near the band-gap^24,25^. It has been observed that the low-energy excitons have changed as a function of temperature, however it requires high-energy bands to understand the low-energy excitations. Since there has been no direct experimental techniques to simultaneously probe high-energy and low-energy bands and how high-energy bands affect low-energy properties, origins of low-energy properties remain hotly debated.

Whilst the electronic properties of bulk-like, layered MoS_2_ were investigated several decades ago^26–28^, the properties of bulk-like, layered materials, such as MoS_2_, in comparison to monolayers, are far from understood and shall not be dismissed. In particular, bulk-like materials are necessary to understand quantum confinement effects, and an electronic transition to a low-dimensionality due to an interplay of interlayer and intralayer electronic correlations could have significant effects. Indeed, further studies have shown that such low-energy properties dramatically change with thickness, from a monolayer toward a bulk-like, layered material, such as the enhancement of spin-polarisation from single-layer to bulk WS_2_^29^, the screening of Coulomb correlations from two-dimensional graphene to bulk-like, layered graphite^30^, and the recent discovery of unconventional superconductivity in graphene superlattices^31^. This raises the fundamental question on the role of electronic, interlayer and intralayer correlations in two-dimensional systems. It is therefore crucial to measure the temperature-dependent and polarization-dependent complex dielectric and loss functions of such bulk-like, layered systems at energies beyond the optical regime.

In all previous studies, the optical properties of TMDCs, particularly for above 3–4 eV, were mainly measured using reflectance or energy loss spectroscopies or a combination of these techniques^28,32–34^. While these techniques are useful, they have fundamental limitations, which lead to inaccurate optical spectra for the following reasons. First, in order to obtain the complex dielectric function and loss function from reflectance and energy loss spectroscopy measurements, one has to perform Kramers–Kronig transformations (KKT), which requires a much broader energy range than the measurement capabilities in order to achieve a stable KKT^35^. However, all existing optical measurements on TMDCs were performed in a limited energy range, therefore the data analysis required extrapolative assumptions of spectra for energies beyond the measured spectral range, which may result in inaccurate low-energy optical spectra. One of the fundamental characteristics resulting from KKT is that any change, even relatively small ones, in the high-energy spectrum would significantly influence the low-energy spectrum^36–38^. Therefore, the limitations on energy measurements and theoretical assumptions made for the high-energy spectra that were temperature-independent and doping-independent, result in unstable KKT, which yields inaccurate complex dielectric functions and loss functions^35–37^. Second, while optical reflectivity, which is a photon-in photon out technique, is bulk sensitive, electron loss spectroscopies are very surface sensitive. Therefore, the normalization and combination of spectra with different penetration depths lead to another discrepancy. Third, since most bulk TMDC systems are actually insulating, electron-loss spectroscopies face so-called charging problems, which then lead to inaccurate spectra and significant broadening and shifting of peaks. Therefore, there was no reliable experimental data of high-energy bands on TMDCs.

In this article, we address this fundamental problem and surprisingly discover unconventional, many-body soft X-ray correlated-plasmons with low-loss in bulk, single crystal MoS_2_ using a new methodology. Interestingly, upon cooling from 400 to 40 K, we find two electronic transitions; a sharp transition from three-dimensional-like to two-dimensional-like system at ~150 K, and a further enhancement of strong electronic correlations at ~100 K, accompanied with significantly modified low-energy excitons. At higher temperatures from 400 K down to 150 K, a soft X-ray correlated-plasmon with low-loss and three-dimensional character is observed at ~35 eV. Surprisingly, Mo *p*–S *d* hybridizations are isotropic between the in-plane and out-of-plane directions, revealing strong interlayer and intralayer correlations yielding a three-dimensional-like system. Upon cooling below 150 K, in-plane (out-of-plane) Mo *p*–S *d* hybridizations become enhanced (reduced), revealing a two-dimensional-like system within the bulk-like system. Further cooling below 100 K, anomalous spectral-weight transfer occurs from high-energies (>3.4 eV) to low-energies bands (≤3.4 eV) revealing strong electronic correlations. As a result of the interplay between strong electronic, interlayer and intralayer correlations, the high-energy bands play a crucial role in determining low-energy properties such as excitons, which become dramatically enhanced and sharpened and their peak energies are blue-shifted.

These direct observations are made using a newly developed experimental technique at the Soft X-ray-Ultraviolet (SUV) beamline of the Singapore Synchrotron Light Source that is capable of combining and analyze Mueller-Matrix spectroscopic ellipsometry, soft X-ray reflectivity, and X-ray absorption spectroscopy measurements as a function of temperature and polarizations in a broad energy range, from 0.6 to 1500 eV. The Mueller-Matrix spectroscopic ellipsometry, which is a self-normalized technique, directly probes the complex dielectric function, loss function, and reflectivity simultaneously in the energy range from 0.6 to 5.5 eV without the need for KKT^37,38^, while the soft X-ray reflectivity covers the energy range from 3.5 to 1500 eV^39^. Since both techniques are photon-in photon-out measurements and can cover an energy range from the infrared to the soft X-ray with high-energy resolution, the combination of these techniques leads to a stable KKT.

## Results and discussion

**Fig. 1:**
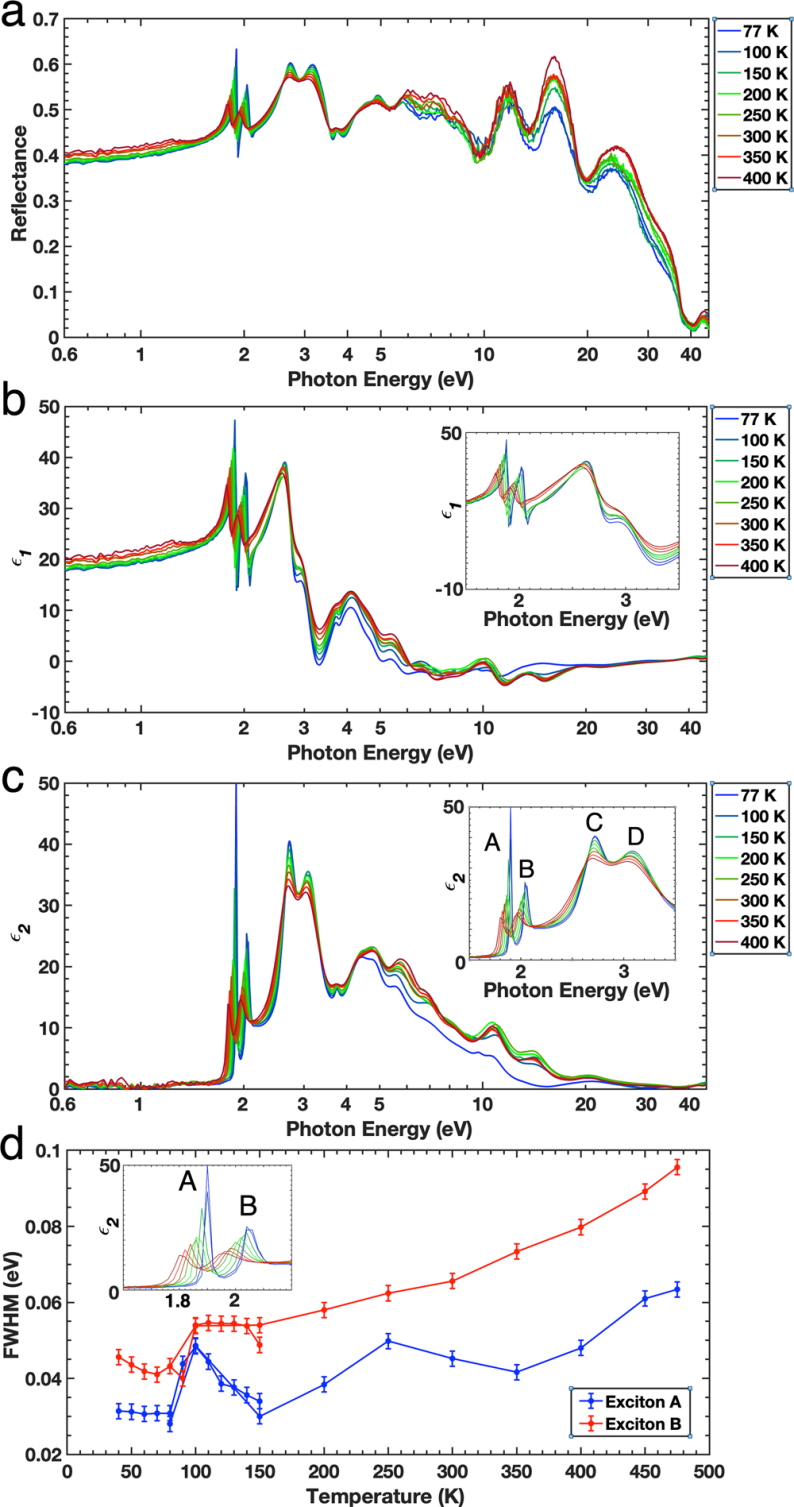
Reflectance measurements and complex dielectric function of MoS_2_ as a function of temperature. **a** Measurements of the reflectance of the bulk MoS_2_ from the near-infrared (0.6 eV) up to soft X-ray (45 eV) using the SUV beamline at SSLS, log scale. The **b** real (*ε*_1_) part and **c** imaginary (*ε*_2_) part of the complex dielectric function of MoS_2_ from spectroscopic ellipsometry and soft X-ray reflectance, log scale. **d** The FWHM of excitons A and B as a function of temperature showing significant change. Error bars are calculated using a 2 meV shift. Insets: Magnified view of complex dielectric function of low-energy excitons from **b** and **c** on a linear scale.

Figure 1 shows reflectance measurements and the complex dielectric function of MoS_2_, measured at selected photon energy ranges from 0.6 to 45.0 eV and as a function of temperature, taken using a combination of the Soft X-ray reflectivity and Mueller-Matrix spectroscopic ellipsometry (see Supplementary Figs. 1 and 2 for details). We also implement a stabilized normalization procedure in which the low energy spectra of the Soft X-ray reflectivity measurements are self-normalizing via spectroscopic ellipsometry and the high-energy spectra are normalized using the low energy spectra and the tabulated values at the high-energy end of the spectrum, yielding a stabilized KKT across all temperatures^37,40^. Utilising this method also shows the significant impact that higher energy measurements have on the determination of the complex dielectric function at lower energies (See Supplementary Fig. 3) and that the complex dielectric function is significantly underestimated near the limits of spectral measurements.

Figure 1a shows surprisingly strong temperature-dependent MoS_2_ reflectivity in a broad energy range, which directly reveals strong electronic correlations in MoS_2_. The real *ε*_1_ (Fig. 1b) and imaginary *ε*_2_ (Fig. 1c) parts of the complex dielectric function of MoS_2_ show a strong temperature dependence. For further clarity, Supplementary Fig. 2a shows the linear plot of the reflectance and Supplementary Figs. 2b and 1c show the linear plot of the complex dielectric function in order to highlight all the features over the wide energy range. The refractive index, *n*, and the absorption coefficient, *k*, are also shown as a function of temperature in Supplementary Fig. 4.

Below 3.4 eV, *ε*_1_ and *ε*_2_ show the presence of the four excitonic features that are commonly labelled A–D^21^ (see inset of Fig. 1b, c, respectively). We find two electronic transitions, at ~150 and ~100 K. As the temperature decreases from 475 to 40 K, the intensity of the low-energy exciton peaks, A and B, become enhanced and their peak energies are blue-shifted (see also Supplementary Fig. 5 for details). Interestingly, the full-width at half-maximum (FWHM) of excitons A and B sharpens significantly by over a factor of 2, from ~60 to ~30 meV and ~95 to ~45 meV, respectively (Fig. 1d). For reproducibility and details, we perform the experiments twice, i.e., the broad range of temperatures initially measured and the detailed temperature measurements that followed. There is a clear difference in the effect of temperature on the FWHM for both excitons with the electronic transitions observed at ~150 and 100 K. As the MoS_2_ is cooled from 400 to 150 K, the FWHM of excitonic peak A decreases, with some fluctuations suggesting the existence of competing interactions. Further cooling from 150 to 100 K, results in the FWHM broadening significantly and then from 100 to 40 K, it narrows sharply again. A similar, less intense event also occurs around 350 K due to the slight increase in the FWHM of exciton A from 350 to 250 K before a decrease down to 150 K. The FWHM of excitonic peak B steadily decreases from 400 to 150 K, and remains nearly temperature independent between 150 K and 100 K, before it suddenly sharpens below 100 K. The FWHM of the excitons are directly linked to their binding energies and life-times, thus there must be some kind of energetic event that occurs, particularly below 350 and 150 K, that causes the low-energy excitons to become unstable (broadening indicates reduced life-times and lowered binding energies^35,41^) and then to re-stabilise rapidly below 100 K. As discussed later, it is found that the temperature dependent low-energy excitons are determined not only by the spectral weight of higher-energy bands, but also the dimensionalities of the material, i.e., whether they are electronically two-dimensional or three-dimensional, particularly the unconventional soft X-ray correlated-plasmons.

An important aspect highlighted by the extended complex dielectric function is the various changes in spectral weight seen across the spectral region. For high-energies above 3.4 eV, interestingly, we observe four significant peaks at higher energies of ~5, ~6, ~11, and ~15 eV that are very strongly temperature-dependent (Fig. 1). The most startling result is drastic changes of the complex dielectric function above 13 eV with two transition temperatures, ~150 and ~100 K. In contrast, below 3.4 eV, the lower temperatures dominate the amplitude, whereas the higher temperatures show much broader peaks (See Supplementary Fig. 3). Above 8 eV the mid-range temperatures of 200 and 250 K dominate the spectral weight whilst the hotter temperatures lose spectral weight.

**Fig. 2:**
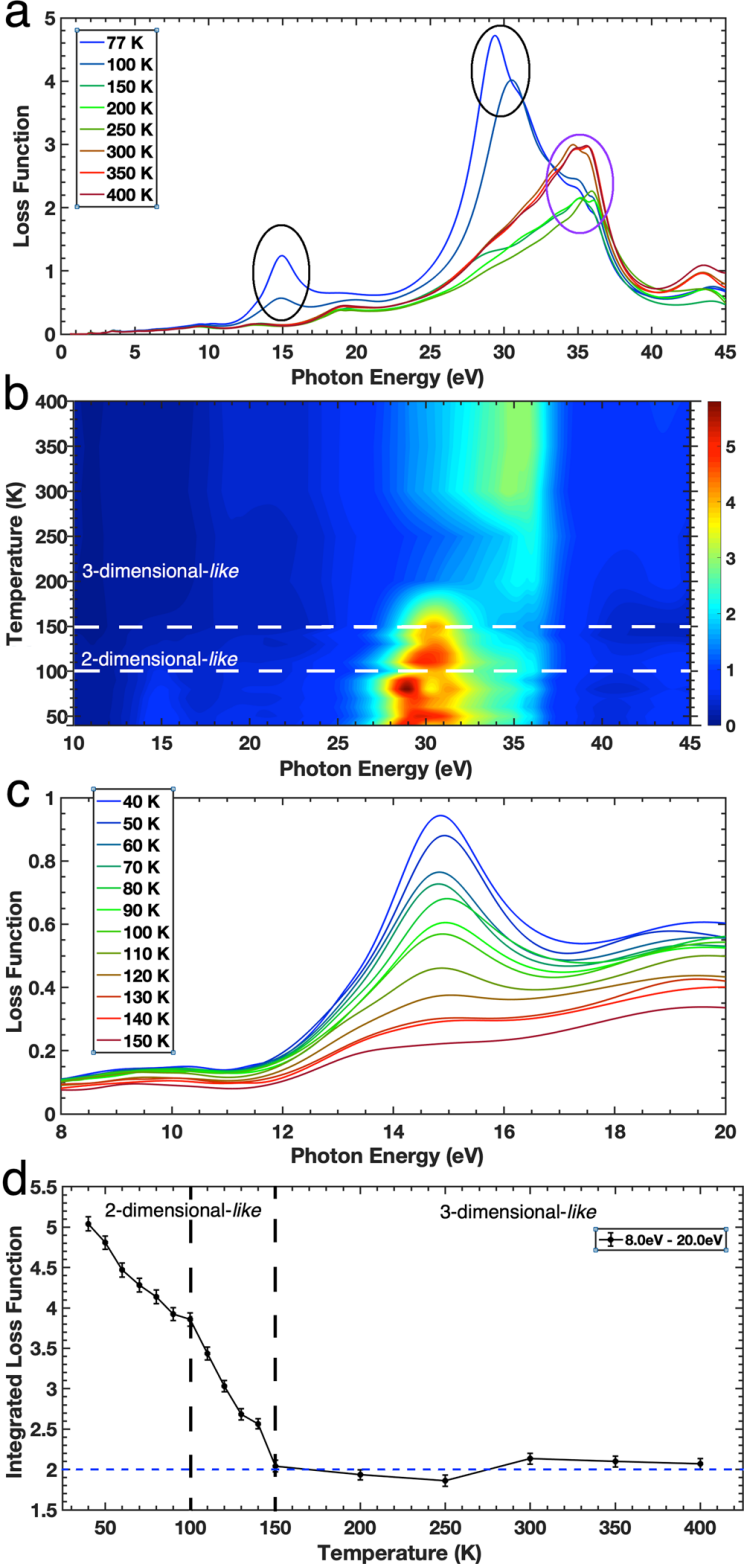
Loss function and spectral weight of bulk MoS_2_. **a** The loss function of MoS_2_ as a function of temperature from 0.6 to 45 eV. The soft X-ray correlated-plasmonic peaks are circled, i.e., black and purple circles are for low-temperature 2-dimensional-like and high-temperature 3-dimensional-like soft X-ray correlated-plasmons, respectively. **b** Contour plot of loss function shows transitions at 150 and 100 K. Note that vertical dashed lines represent the transition temperatures. **c** Detailed measurements of the loss function of MoS_2_ from 8 to 20 eV in the temperature range of 40–150 K at 10 K intervals. **d** Evolution of the soft X-ray correlated-plasmon at ~15 eV as a function of temperature. Note that the dashed vertical lines represent the transition temperatures and dotted horizontal line is a background level. Error bars are calculated using a 20 meV shift.

Our main observation is unconventional soft X-ray correlated-plasmons, one at ~35 eV observed even from high temperature of well above room temperature and a pair at ~15 and ~30 eV observed below ~150 K as seen in the loss function of MoS_2_ shown in Fig. 2 (highlighted in Fig. 2a, b). The loss function is directly calculated from the complex dielectric function by

1 $${{{{{\rm{LF}}}}}}=-{{{{{\rm{Im}}}}}}(1/\varepsilon )=-{{{{{\rm{Im}}}}}}(1/({\varepsilon }_{1}+i{\varepsilon }_{2})),$$

across the entire spectral region for all the temperatures measured. It is very sensitive for any plasmonic activity within the material. For photons of energy <1 keV, the momentum transfer, *q*, is finite but approaches zero because it is much less than the crystal momentum. In this limit, the distinction between the longitudinal and transverse *ε*(*ω*) vanishes i.e.,

2 $$\mathop{{{{{\mathrm{lim}}}}}}\limits_{q\to 0}{\varepsilon }_{l}(q,\omega )={\varepsilon }_{t}(q,\omega ),$$

which allows spectroscopic ellipsometry and soft X-ray reflectivity measurements to probe both the optical and plasmonic properties in the low-*q* limit^8,41^.

It is clear that the loss function of MoS_2_ at the lower temperatures of 150 and 100 K is significantly different from that at higher temperatures. At 400 K, we observe a loss function peak at ~35 eV that persists down to 40 K. The soft X-ray peak at ~35 eV in the loss function shows unique characteristics of correlated-plasmons^11^, in which the peak only occurs in the loss function and is not observable in *ε*_2_ (Fig. 1c), while *ε*_1_ is nearly zero but remains positive (Fig. 1b), and the reflectivity show a local minimum (Fig. 1a). These properties are shown more clearly in Supplementary Fig. 4. This reveals that the ~35 eV peak is a new unconventional plasmon in the soft X-ray regime of the strongly correlated-material MoS_2_, namely a soft X-ray correlated-plasmon. Having a positive *ε*_1_ reveals very weak screening, therefore the Coulomb correlations are strong. The loss function remains temperature independent upon cooling from 400 to 300 K. The intensity of the ~35 eV soft X-ray correlated-plasmon reduces as MoS_2_ is cooled to 150 K. This is unusual as, typically, plasmons should become stronger at lower temperatures.

Interestingly, upon lowering the temperature below 150 K, a pair of soft X-ray plasmons occurs at ~15 and ~30 eV and coherently gets stronger upon cooling. Since these ~15 and ~30 eV peaks only occur in the loss function and are not observable in *ε*_2_ (Fig. 1c) and *ε*_1_ is nearly zero but still positive (Fig. 1b), these are a pair of soft X-ray correlated-plasmons at low temperature and the Coulomb correlations are getting stronger upon cooling. The soft X-ray correlated-plasmons are close to the density of states where the S 3*s* hybridizes with the Mo 4*d* states lying between the valence bands and the core levels (namely semi-core states)^27,28,42,43^. Furthermore, previous theoretical calculations of the energy electron loss function have suggested strong plasmonic character at this energy^44^. Subsequently, hybridizations in the semi-core play a role in generating the soft X-ray correlated-plasmons.

In Fig. 2c, we show the temperature dependent soft X-ray correlated-plasmon at ~15 eV because its structure is less complex and low loss as *ε*_2_ is nearly zero. Details measurements of the complex dielectric function between 0.6 and 45 eV within the temperature range 40–150 K at 10 K intervals are then undertaken to measure the rise of the cold soft X-ray correlated-plasmon. As the temperature decreases below 150 K, the soft X-ray plasmon shows up and its intensity start to increase steadily with another transition at ~100 K (Fig. 2d). The appearance of the soft X-ray correlated-plasmons clearly has significant effects on the electronic structures of the material and this also nicely explains the destabilisation of the low-energy excitons seen in Fig. 1d with different transition temperatures. It is revealed that the formation of the soft X-ray correlated-plasmons has a direct impact in stabilising the whole system. However, they are not a result of any structural changes as the crystal structure of MoS_2_ is stable from 773 K down to 10 K and no structural transitions occur^45,46^. In order to shed some light on the existence of these soft X-ray correlated-plasmons and why they decrease and increase in intensities as the temperature is lowered, we examine the spectral weight transfer and hybridizations through temperature-dependent and polarization-dependent soft X-ray absorption spectroscopy, and extended band-structure and complex dielectric function calculations.

**Fig. 3:**
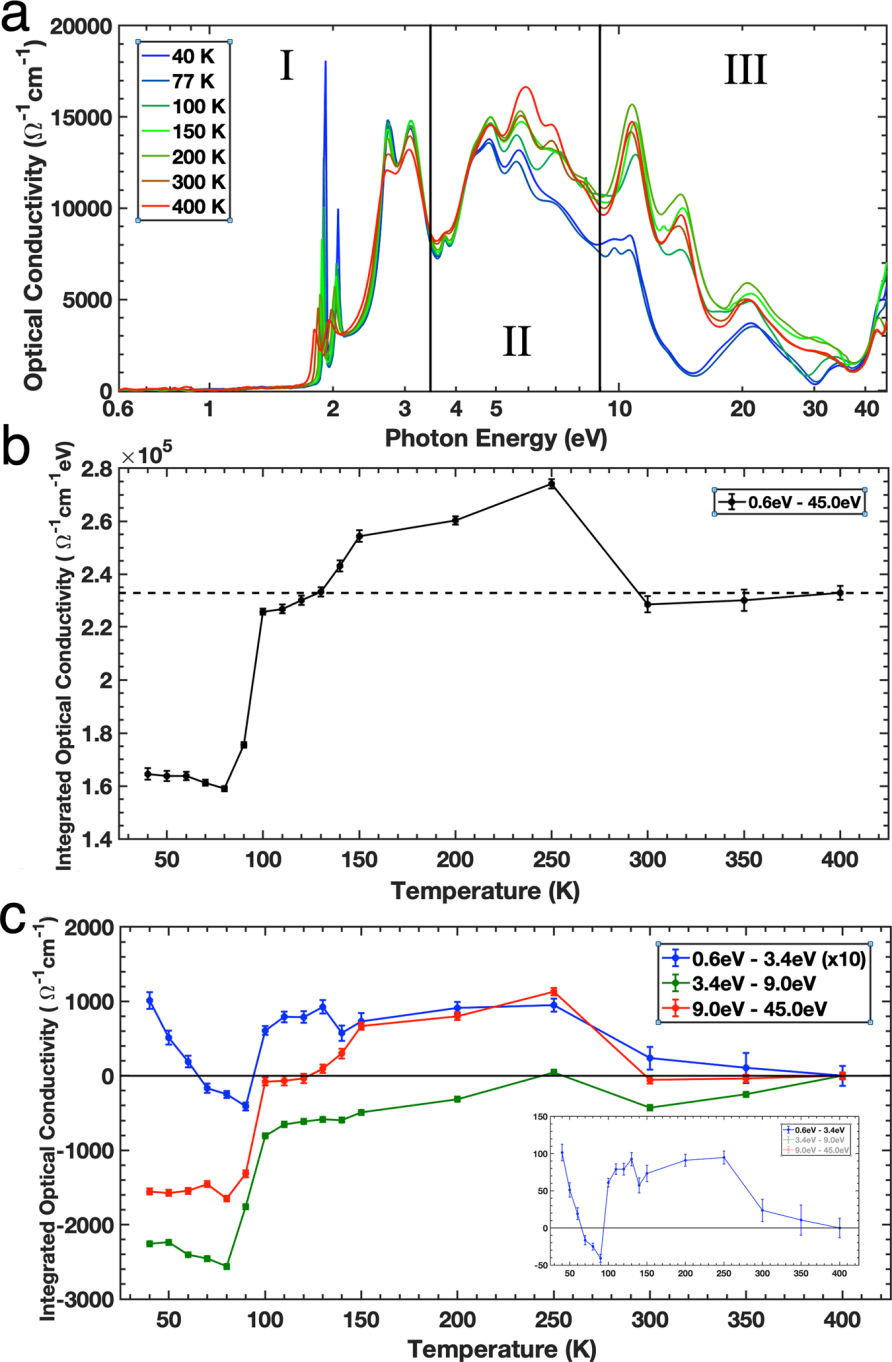
Spectral weight transfer of MoS_2_. **a** The optical conductivity of MoS_2_ of selected temperatures divided into three spectral regions. **b** The optical conductivity integrated over the whole spectrum as a function of temperature. Error bars are calculated using a 20 meV shift of spectral divide. **c** The relative change in integrated optical conductivity of the three different spectral regions as the sample is cooled from 400 to 40 K. Region I has been magnified for clarity. Inset: Original values of Region I. Error bars are calculated using a 20 meV shift of spectral divide.

Figure 3a shows the optical conductivity of the MoS_2_ for a selected number of temperatures. By analysing the transfer of the spectral weight of the optical conductivity, *σ*_1_ between 0.6 and 45 eV as a function of temperature, we determine the origin of these high-energy plasmons at low temperatures. For this analysis, the measured spectral range is divided into three spectral regions as follows; I: 0.6–3.4 eV, II: 3.4–9.0 eV and III: 9.0–45.0 eV, with error bars are calculated based on 20 meV shift on either side of spectral divide.

The integration of the optical conductivity is related to the free-charge carrier density, *n*_e_, via the charge conserving *f*-sum rule:

3 $${\int }_{0}^{\infty }{\sigma }_{1}(\omega ){{{{{\mathrm{d}}}}}}\omega =\pi {n}_{{{{{{\mathrm{e}}}}}}}{e}^{2}/2{m}_{{{{{{\mathrm{e}}}}}}}$$

where *e* is the elementary charge and *m*_e_ is the electron mass^37,41^. Therefore, the integration of a part of the spectral region *W* between *E*_1_ and *E*_2_, given by:

4 $$W={\int }_{{{{{{{\mathrm{E}}}}}}}_{1}}^{{{{{{{\mathrm{E}}}}}}}_{2}}{\sigma }_{1}(E){{{{{\mathrm{d}}}}}}E$$

is proportional to the number of free charge carriers within that spectral region. By analyzing the change of *W* with temperature we also gain a unique insight into the behaviour of the free charge carriers as the MoS_2_ sample is cooled.

The total spectral weight, *W*_tot_, of the MoS_2_ across the measured spectral range of 0.6–45.0 eV is shown in Fig. 3b as a function of temperature. Upon cooling, *W*_tot_ is nearly constant from 400 to 300 K, increases generally from 300 to 150 K, then decreases slowly below 150 K before a rapid drop-off below 100 K. This indicates that there are more electrons with energies from 0.6 to 45.0 eV between 300 and 150 K than at any other temperature, which means that above and below that temperature range, the number of electrons with energies outside of this spectral range is increasing. This is reversed below 150 K and the spectral weight decreases dramatically below 100 K. The extra energy gained or lost in such a broad energy range comes from the potential energy of electron–electron correlations yielding strong Coulomb correlations below 100 K^36^. Interestingly, the soft X-ray correlated-plasmons seen in Fig. 2 are observed to have different transition temperatures coinciding with the rise or fall in electron conductivity and thus electron density, as seen in Fig. 2b. These shifts in spectral weight involve the loss or gain of a few tens of eV and thus cannot be explained by thermal activation alone as the energies associated with temperatures below 500 K are too small (<43 meV). Furthermore, they also cannot be explained by electron-phonon interactions as in MoS_2_, the energy gained by these interactions is only 9 cm^−1^ or 1.1 meV^47^. Similarly, exciton-plasmon interactions can also be discounted as the coupling strength is only 53 meV^48^.

We now discuss the connection between the soft X-ray correlated-plasmons and spectral weight of each region (*W*_I_, *W*_II_, and *W*_III_ for regions I, II, and III, respectively). Figure 3c shows the relative change in *W* of each of the three spectral regions from Fig. 3a as the sample is cooled from 400 to 40 K. As temperature decreases from 400 to 300 K, *W*_III_ is nearly constant, while *W*_I_ and *W*_II_ show only small changes. At this temperature range, we observe the soft X-ray correlated-plasmon at 35 eV and its intensity is nearly constant. As the temperature further decreases from 300 to 250 K, there is a sharp increase in all regions followed by a small and gradual decrease from 250 to 150 K. The sharp increase between 300 and 250 K is the result of an increased number of electrons with energies that occupy these spectral ranges. As this occurs across all of the spectral regions, this reveals that the extra electrons are from above 45 eV. This means that we are seeing a drastic loss in electron energy of the order of several tens of eV, which is far too large to be accounted for by thermal changes, or by electron–phonon or electron–plasmon interactions as stated earlier^47,48^. A loss of energy this large comes from the loss of electrons from the 35 eV soft X-ray correlated-plasmon, resulting in the reduction of its intensity seen in Fig. 2a. This is accompanied with steadily enhancing, sharpening, and blue-shifting of low energy excitons (Fig. 1b–d and see also Supplementary Fig. 5).

The spectral weight of the low-energy excitons represented in *W*_I_ increases from 150 to 100 K (see also Fig. 3a). Conversely, *W*_III_ decreases upon cooling whereas W_II_ remains unaffected. We are seeing a transfer of spectral weight from region III to region I, whereby electrons from the high-energy bands of region III lose energy and contribute to the enhancement of the low-energy excitons. The fact that these low-energy excitons depend on such high-energy bands is a surprising result. The loss of spectral weight in region III upon cooling down to 100 K is also a result of an increase in electron energy due to the increasing number of electron–electron correlations. As the MoS_2_ cools, the electronic screening steadily reduces, giving rise to more electron-electron correlations across the material. The excitonic features A–D blue-shift and sharpen with cooling, despite the fact that there is a loss in spectral weight from the higher energy regions to region 1. This means that there are more electrons with energies between 0.6 and 3.4 eV at lower temperatures than there were at higher temperatures and results in the magnitude of the excitonic peaks increasing. The blue-shifting of the peaks (Supplementary Fig. 5) is a result of the increasing potential energy due to the reduced electronic screening and steadily increasing electron–electron correlations^49^.

Further cooling below 100 K, results in the drastic reduction of spectral weight in all regions are due to the formation of the soft X-ray correlated-plasmons at ~15 and ~30 eV. The loss in spectral weight at lower temperatures from the *W*_II_ and *W*_III_ regions represents a significant shift in electron density from this spectral range to even higher energies (above 45.0 eV) and to low energies (below 3.4 eV). The increase in electron energy of the order of 10’s of eV comes from long-range, high-energy electron–electron correlations, which are now prominent due to weakening electronic screening upon cooling. These electron–electron correlations form the soft X-ray correlated-plasmons at ~15 and ~30 eV in Fig. 2. It is clear that the massive spectral weight transfer over a broad energy range, which is a fingerprint of strong electronic correlations, is caused by the formation and reduction of the soft X-ray correlated-plasmons at 15, 30, and 35 eV. Below 100 K, the spectral weight of regions II and III remain relatively constant whilst region I shows an increase due to the continuing enhancement of the low-energy excitons down to 40 K.

It is worth mentioning that the values of *n* near the energy of the plasmons are nearly zero but remain positive, revealing that these are unconventional correlated-plasmons in the soft X-ray region (see also Supplementary Fig. 6). Note that correlated-plasmons in the near-IR and optical regions have previously been found within low-dimensional Niobates further support that unconventional soft X-ray correlated-plasmons in MoS_2_ are due to strong electronic correlations^50^. The corresponding values of *k* are low, e.g. *k* ~0.2 at 30 eV, revealing low-loss correlated plasmons. This is very unusual because such high energies typically contained a considerable number of inter-band transitions, therefore one might expect to find a reasonably high absorption. The soft X-ray correlated-plasmons can occur in materials with a low free-charge density and arise from the collective oscillation of quasi-local (or correlated) electrons, which means they can have more than one plasmon energy. Correlated-plasmons are a new type of plasmon driven by electronic correlations and framing the unconventional correlated-plasmons as collective electron gas effects would not be accurate^50,51^. For the case of MoS_2_, by assuming a free electron gas, the equation for plasma frequency applies:

5 $${\omega }_{{{{{{\mathrm{p}}}}}}}={E}_{{{{{{\mathrm{p}}}}}}}/\hslash =\sqrt{\frac{n{e}^{2}}{{m}_{{{{{{\mathrm{e}}}}}}}{\varepsilon }_{0}}},$$

where *n* is the electron density, *m*_e_ is the electron mass, *ε*_0_ is the permittivity of free space, *e* is the elementary charge, $\hslash$ is the reduced Planck’s constant, and *ω*_p_ and *E*_p_ are the plasmon frequency and energy respectively. The 15 eV plasmon would, in conventional terms, have an electron density of 1.632 × 10^23^ cm^−1^. MoS_2_ has a mass density of 5.06 g/cm and a molar mass of 160.07 g/mol, so assuming a perfect crystal, the molecular density is 1.904 × 10^22^ cm^−1^. This would mean that about 8.6 free electrons per molecule would be needed to generate the 15 eV plasmon. For the 30 eV plasmon this would be 34.3 free electrons per molecule and the 35 eV plasmon would need 46.7 free electrons per molecule. This is clearly not feasible and further supports that these soft X-ray correlated-plasmons are quantum oscillations of correlated-electrons.

**Fig. 4:**
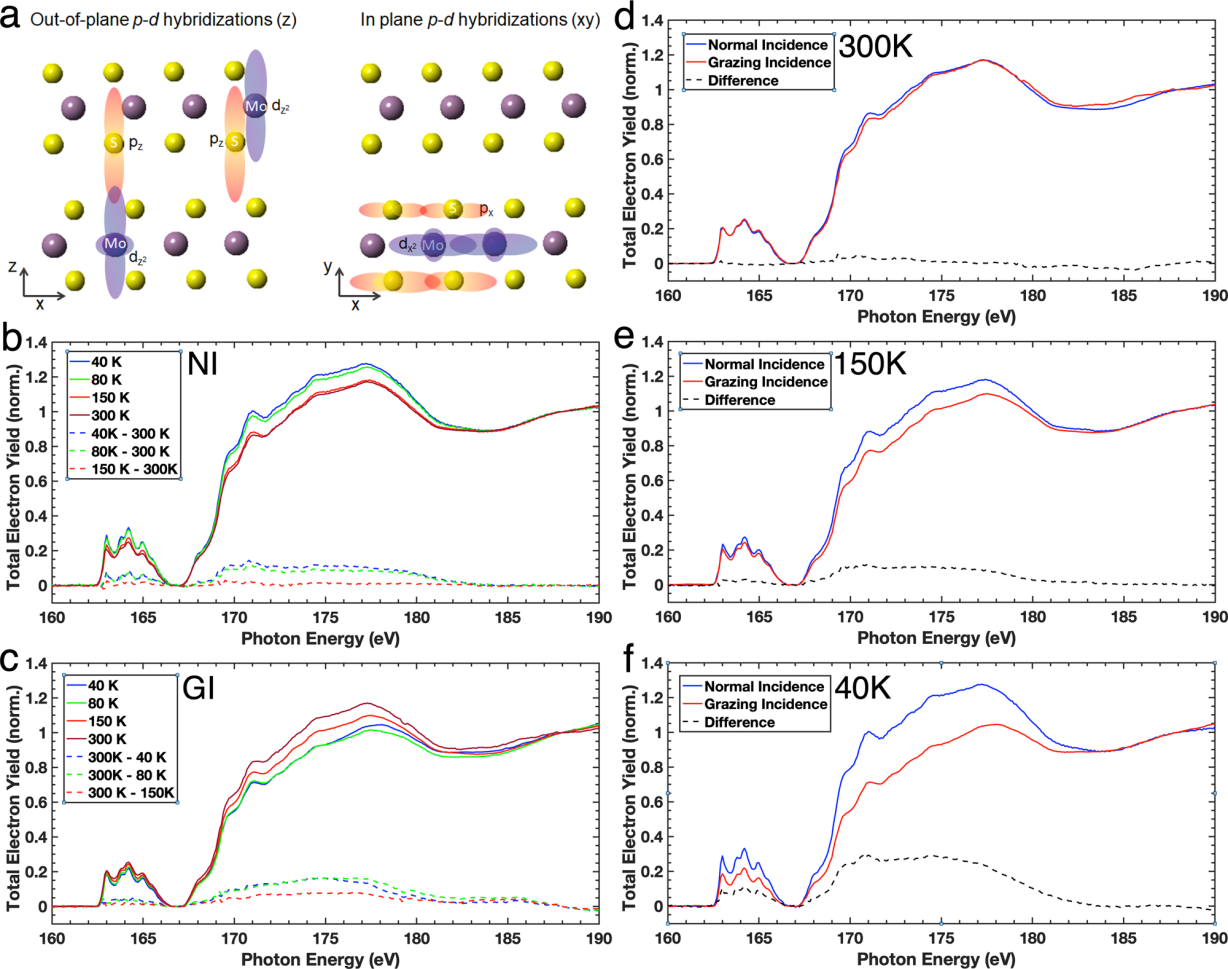
Temperature-dependent X-ray absorption spectroscopy (XAS) spectra at S *L*_2,3_ edges and their differences with respect to 300 K data on MoS_2_. **a** A schematic diagram of in-plane and out-of-plane *p*–*d* hybridisations in MoS_2_. XAS spectra when the sample is at **b** normal (NI) and **c** grazing incidence (GI) angles, respectively, as a function of temperature. **d**–**f** Comparison of normal and grazing incidence XAS spectra for 300 K, 150 K, and 40 K, respectively.

To further reveal the origin of the soft X-ray correlated-plasmons and its transitions, we perform temperature-dependent soft X-ray absorption spectroscopy (XAS) at S *L*_2,3_ edges of MoS_2_ as shown in Fig. 4. The XAS measurements are carried out using linear polarizations under two different geometries: normal incidence (NI), in which the photon polarization was parallel with the in-plane direction, and grazing incidence (GI), in which the photon polarization was in the out-of-plane direction. In-plane and out-of-plane polarization dependent XAS are common techniques to study inter-layer and intra-layer electronic coupling and dimensionality, and has been used extensively, especially in strongly correlated electron systems such as La_2-x_Ba_x_CuO_4_ and Sr_14_Cu_24_O_41_^52,53^, which show strong polarisation dependence along the **a**, **b**, and **c** directions. Polarisation-dependent XAS reveals the dimensionality of the electronic structure of such materials. The strengthening of in-plane (intra-layer) electronic coupling and weakening of out-of-plane (inter-layer) electronic coupling are a clear indication that the system is electronically becoming more two-dimensional-like. The XAS data are normalized off-resonance, below and above the edges. The S *L*_3,2_-edge consists of two main spectral features: the pre-edge spectra within 162–167 eV corresponding to the transition from S 2*p*_j=3/2,1/2_ to S 3*p*-Mo 4*d* hybridized states, and the larger post-edge spectral features around 167–185 eV, which are attributed to the S 2*p*_3/2,1/2_ → S 3*d* (hybridized with Mo 5*p* states) transition^43^. These *p*–*d* hybridizations are illustrated in Fig. 4a for both the in-plane and out-of-plane directions within the layered MoS_2_. We note that the out-of-plane *p*–*d* hybridizations consist of interlayer and intralayer correlations, while the in-plane *p*–*d* hybridizations mostly consist of intralayer correlations.

In Fig. 4b, the temperature dependent XAS at NI, which probes the in-plane *p*–*d* hybridizations, is shown. Upon cooling, the spectral weight increases below 150 K, which reveals that the *p*–*d* hybridizations within the layer become enhanced as the MoS_2_ is cooled. In contrast, this is not the case for the temperature dependent XAS at GI as shown in Fig. 4c. This probes the out-of-plane *p*–*d* hybridizations and, upon cooling, the spectral weight decreases below 150 K. This shows that as the temperature decreases, the *p*–*d* hybridizations of interlayer and intralayers decrease in the **z**-direction, resulting in a two-dimensional-like system at low temperatures.

Next, we compare the polarization dependent XAS taken at different temperatures (Fig. 4d–f) under these two different geometries. At 300 K (Fig. 4d), the XAS spectra for NI and GI are similar, revealing that the in-plane and out-of-plane *p*–*d* hybridizations are isotropic at higher temperatures. This isotropic nature is a surprising result, revealing strong interlayer and intralayer correlations along the **z**-direction, which are as strong as the intralayer correlations in the **xy**-plane (Fig. 4a), yielding a three-dimensional-like system at high temperature and thus the soft X-ray correlated-plasmon has three-dimensional-like character. Upon cooling to 150 K and then 40 K (Fig. 4e, f, respectively), there is a significant change in the absorption intensity between the NI and GI spectra. The *p*–*d* hybridizations are now highly anisotropic in which the intralayer correlation in the **xy**-plane (or in-plane *p*–*d* hybridizations) are stronger than the interlayer and intralayer correlations in the **z**-direction (or out-of-plane *p*–*d* hybridizations), yielding a two-dimensional-like system. The anisotropy of the *p*–*d* hybridizations weakens (enhances) the electronic screening within (across) the layers, respectively and leads to the suppression of the three-dimensional-like soft X-ray correlated-plasmon at ~35 eV and the formation of the soft X-ray correlated-plasmons at ~15 and ~30 eV, which instead, have a two-dimensional electronic character below 150 K. We note that while the interlayer and intralayer atomic coupling gets stronger upon cooling, the electronic correlations behave differently depending on the hybridisations of the electron orbitals of the material^47^. This is because the degree of *p*–*d* hybridizations are determined through bond angle and length between and within the S–Mo–S layers. As Fig. 4a shows, hybridizations get stronger because the angle between atoms is approaching 180° (or away from 90°). We note that Raman spectroscopy shows significant blue-shifting with decreasing temperature, meaning that atoms within layers become closer and have stronger phonon coupling^47^, which then further enhances the out-of-plane *p*–*d* hybridizations.

Further cooling below 100 K, Coulomb correlations get stronger as well as the intralayer correlation enhancing the soft X-ray correlated-plasmons, as revealed from spectral-weight transfer (Fig. 3c). The transitions from the three-dimensional-like to the two-dimensional-like system which yield the unconventional soft X-ray correlated-plasmons are quantum confinement effects in bulk-like MoS_2_. The high-energy bands, interlayer and intralayer correlations, and unconventional soft X-ray correlated-plasmons determine low-energy properties such as excitons.

**Fig. 5:**
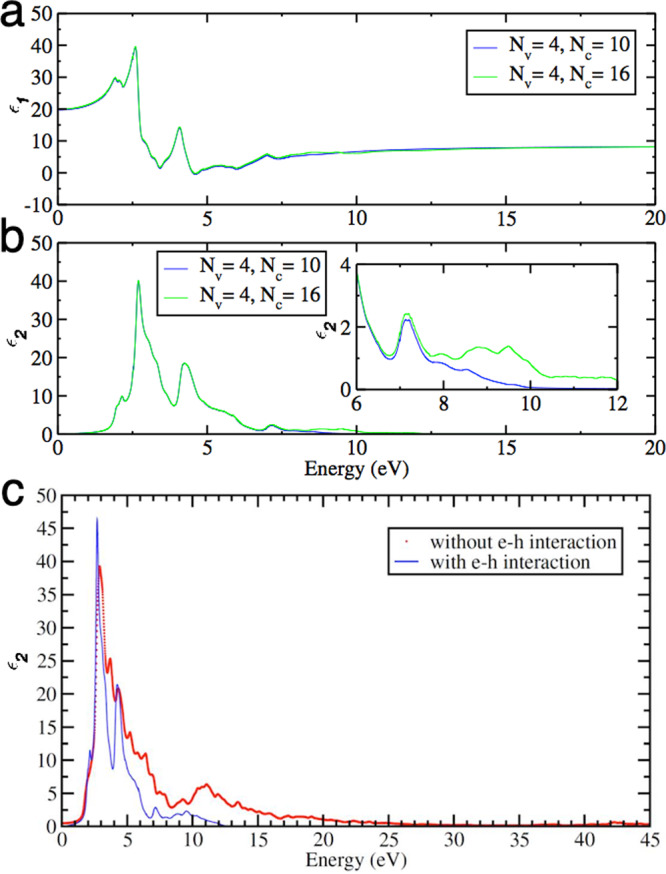
High-energy dielectric function calculations. The **a** real and **b** imaginary parts of the complex dielectric function calculated with four valence bands and 10 (blue) and 16 (green) conduction bands. **c** Calculations of the imaginary part of the complex dielectric function with and without electron–hole interactions.

To further reveal the roles of electron–electron and electron–hole interactions and high-energy bands in MoS_2_, Fig. 5a, b show *ε*_1_ and *ε*_2_ calculations in a broad energy range, respectively. To understand the impact of higher energy bands, the number of valence bands used for these calculations is *N*_v_ = 4, while the number of conduction bands used are varied, *N*_c_ = 10 (blue lines) and *N*_c_ = 16 (green lines). Clearly, an increased number of high-energy conduction bands results in more inter-band transitions yielding an increase in calculated *ε*_2_ at higher energies (Fig. 5b), which are qualitatively consistent with the experimental data (c.f. Fig. 1c). Interestingly, when the electron–hole interaction is turned on, the calculated *ε*_2_ at high energies reduces dramatically and the spectral weight at high energies push toward low energies (Fig. 5c). This is qualitatively consistent with the low temperature data (77 K) in which spectral weight transfer occurs from high to low energies upon cooling as discussed earlier, with the effects being the enhancement of the excitons, as seen in the low energy part of the calculated *ε*_2_ (Fig. 5c). Furthermore, *ε*_2_ between 12–13 eV does show significant reduction as low as ~2–3 and *ε*_1_ is near zero (as seen in Supplementary Fig. 2c). For further support, the roles of number of valance and conductions bands to the low energy excitations are sown in Supplementary Fig. 7. By combining our experimental results and theoretical calculations, we find that electron–electron and electron–hole interactions, and high-energy bands are important to comprehensively determine the electronic structure and optical properties over a broad energy range. The strength of these interactions, and thus the electronic screening, changes as a function of temperature and the high-energy bands play important role in determining the low-energy excitations, including low-energy excitons in MoS_2_.

This also succinctly explains the behaviour of the FWHM of the low-energy A and B excitons seen in Fig. 1d. When the pair of soft X-ray correlated-plasmons at 15 and 30 eV occurs around 100 K (due to electron–electron correlations and hybridizations), the low-energy excitons are directly affected, as evidenced by the increase and subsequent decrease of the exciton’s FWHM, and thus their lifetime. Similarly, the changes that occur in exciton A from 350 to 250 K are due to the reduction of the 3D-like soft X-ray correlated-plasmons below 350 K. This is very unusual because the energy separations between the soft X-ray correlated-plasmons are as high as ~28 eV. This reveals a strong coupling between the high-energy and low-energy bands as shown through the coupling of the correlated-plasmons and low-energy excitons.

In summary, the unusual 3D character of the electronic structure at room temperature and 3D to 2D-like electronic transitions observed upon cooling in MoS_2_ reveal the importance of the interplay of electronic correlations and *p*–*d* hybridizations between different S–Mo–S layers (interlayer correlations) and within the S–Mo–S layers (intralayer correlations), which are determined by the length and bonding angle degrees of freedom. Such an interplay yields many-body soft X-ray correlated-plasmons with low-loss, which also opens up the applications on the use of transition metal dichalcogenides into plasmonic nanolithography within a broad energy range, from the deep ultraviolet to the soft X-ray. The advantages are that the soft X-ray correlated-plasmons exists even well above room temperature and occur naturally in the bulk form, therefore it simplifies the fabrication and integration processes of plasmonic nanolithography devices^54^. Furthermore, as a function of temperature, one can select different types of correlated-plasmons. Our result shows the importance of strong electronic, interlayer and intralayer correlations in determining the complete optical and electronic structures of strongly-correlated transition metal dichalcogenides.

## Methods

### Sample preparation

The bulk, single crystal MoS_2_ was purchased from HQ Graphene and a single flake with a flat surface was used in both the spectroscopic ellipsometry and reflectivity measurements. In both experiments the sample is heated to high temperatures and left for several hours to remove surface contaminants before any measurements were taken. Characterizations of the crystal can be found in http://www.hqgraphene.com/MoS2.php, which includes X-ray diffraction (XRD), energy dispersive X-ray spectroscopy (EDX), and Raman spectroscopy.

### Muller-matrix spectroscopic ellipsometry

Muller-matrix spectroscopic ellipsometry measurements are carried out using a variable-angle spectroscopic ellipsometer (V-VASE, J.A. Woollam Co.) with a rotating analyser and compensator^37^. Two sets of measurements on bulk MoS_2_ are taken in the energy range of 0.6–5.5 eV whilst the samples are inside an ultra-high vacuum cryostat with a base pressure of 10^−8^ mbar. The sample is cooled to 77 K using liquid Nitrogen and measurements are taken at angles of 68, 70, and 72 degrees, which are limited in range by the UHV windows. The sample is then heated slowly to temperatures of 100, 150, 200, 250, 300, 350, and 400 K. Each of these measurements are taken at a single angle of 70° for each temperature.

The second set of measurements involve the MoS_2_ being cooled to 40 K using liquid Helium and then heated slowly to temperatures of 40, 50, 60, 70, 80, 90, 100, 110, 120, 130, 140, and 150 K. The cooling/heating of the samples is uniform and slow and they are kept at each temperature for 2 h as the measurements are taken. The Muller-matrix spectroscopic ellipsometry directly measures change of amplitude Ψ and phase Δ and from which we obtain the complex dielectric function, loss function, and reflectivity.

### Soft X-ray reflectance measurements

The reflectance measurements are taken at the Soft X-ray-Ultraviolet (SUV) beamline equipped with a unique ultra-high vacuum (UHV) diffractometer of 5-circle geometry, full control of polarization and low temperature capabilities at the Singapore Synchrotron Light Source (SSLS)^39^. The single crystal MoS_2_ is held in a vacuum chamber at 10^−9^ mbar and illuminated with a high-intensity photon beam from the synchrotron radiation. The energy of the photon beam is then tuned from 3.5 to 45 eV and the reflected light is measured by a diode positioned at an angle of 20° with respect to the surface. The data is then normalised by the recorded current of the beam to remove the effects of beam fluctuation during the recording of the data and then also normalised with the throughput of the beam to get the total reflectance of the material.

The temperature of the MoS_2_ was increased from 77 to 400 K via control of the sample holder temperature with a temperature gauge on the sample. The sample is heated slowly to temperatures of 100, 150, 200, 250, 300, 350, and 400 K. The second set of measurements involve the MoS_2_ sample being cooled to 40 K using liquid Helium and then heated slowly to temperatures of 40, 50, 60, 70, 80, 90, 100, 110, 120, 130, 140, and 150 K. The heating of the sample is uniform and slow and they are kept at each temperature for 30 min before the measurements are taken and for 1 h during the measurements.

### Soft X-ray absorption spectroscopy

The temperature-dependent and polarization-dependent soft X-ray absorption spectroscopy (XAS) measurements are carried out at the SUV beamline equipped with a unique ultra-high vacuum (UHV) diffractometer of 5-circle geometry, full control of polarization and low temperature capabilities of SSLS^39^. In particular, the S *L*_3,2_-edge spectra are collected in the total electron yield (TEY) mode in the energy range of 155–195 eV. The MoS_2_ is held in a vacuum chamber at 10^−9^ mbar and illuminated with a high-intensity photon beam from the synchrotron radiation. The sample is subjected to linearly polarized X-ray photons under both normal and grazing incidences providing in-plane and out-of-plane polarizations, respectively. The X-ray photons are then absorbed by the sample and produces electric current, which is counted by a very sensitive current meter. The data are normalized to the incident x-ray beam intensity, then an energy-independent background is subtracted and scaled to get the total electron yield of the sample. The temperature of the MoS_2_ is increased from 40 to 300 K via control of the sample holder temperature with a temperature gauge on the sample. The sample is heated slowly to temperatures of 80, 150, and 300 K.

### Band-structure calculations

The ground state structure, the Kohn-Sham states and the corresponding energies are calculated using density functional theory as implemented in the Quantum ESPRESSO package^55^. A cut-off energy of 40 Ry is used for the wave-function expansion. We employ a fully relativistic pseudopotential to account for core electrons and further to calculate the spin–orbit coupling. The Brillouin zone is sampled using 12 × 12 × 1 **k**-point grid.

The many-body effects are calculated using many-body perturbation theory implemented in the Yambo code. The many-body corrections to the previously calculated Kohn–Sham energies are calculated within G_0_W_0_ approximation to the self-energy. The absorption spectrum, i.e., the complex dielectric function, are then calculated by solving the Bethe–Salpeter equation. As for the calculation of the Bethe–Salpeter matrix, we use the same sample of the Brillouin zone interpolated to larger grid by using double-grid technique. We employ 200 unoccupied bands to build the polarizability.

The size of the matrix that has to be diagonalized to solve Bethe-Salpeter equation is 2 × *N*_v_ × *N*_c_ × *N*_k_, where 2 comes from the spin degree of freedom, *N*_v_ and *N*_c_ are the number of valence and conduction bands, and *N*_k_ is the number of **k**-points in the Brillouin zone. With the symmetry of the system using a double-grid method, we have only 78 inequivalent **k**-points, yet the size of the Bethe–Salpeter matrix can easily reach thousands. The number of valence and conduction bands can be chosen to be small if one is only interested in the visible light region. Since we are interested in calculating the spectrum up to 20 eV, here we use *N*_v_ = 4 and *N*_c_ = 10 and 16.

### Supplementary information

Supplementary Information

## Data Availability

All data that supports the findings of this study are available from the corresponding author upon reasonable request.
