# Supplementary material for: Unravelling strong electronic interlayer and intralayer correlations in a transition metal dichalcogenide
Source: Nat Commun. 2021 Nov 30;12:6980. doi: 10.1038/s41467-021-27182-y (PMC8632915; doi:10.1038/s41467-021-27182-y)
Supplement: Supplementary file 1 — Supplementary Information [file 41467_2021_27182_MOESM1_ESM.pdf]

Unravelling Strong Electronic and Interlayer Correlations in a Transition Metal Dichalcogenide – Supplementary Information

T. J. Whitcher[1,2,3,†], Angga Dito Fauzi[1,2], D. Caozheng[2], X. Chi[2,3], A. Syahroni[4], T. C. Asmara[1], M. B. H. Breese[1,2], A. H. Castro Neto[2,5], A. T. S. Wee[2,5], M. Aziz Majidi[4], and A. Rusydi[1,2,3,5,6,*]

[1]Department of Physics, National University of Singapore, 2 Science Drive 3, Singapore 117576, Singapore

[2]Singapore Synchrotron Light Source, National University of Singapore, 5 Research Link, Singapore 117603, Singapore

[3]Centre for Advanced 2D Materials, National University of Singapore, 2 Science Drive 3, 117546, Singapore

[4]Department of Physics, University of Indonesia, Depok 16424, Indonesia

[5]NUSSNI-NanoCore, National University of Singapore, Singapore 117576, Singapore

[6]NUS Graduate School for Integrative Sciences and Engineering, 117456, Singapore

Correspondence to: [*]andrivo.rusydi@nus.edu.sg or [†]c2dwtj@nus.edu.sg

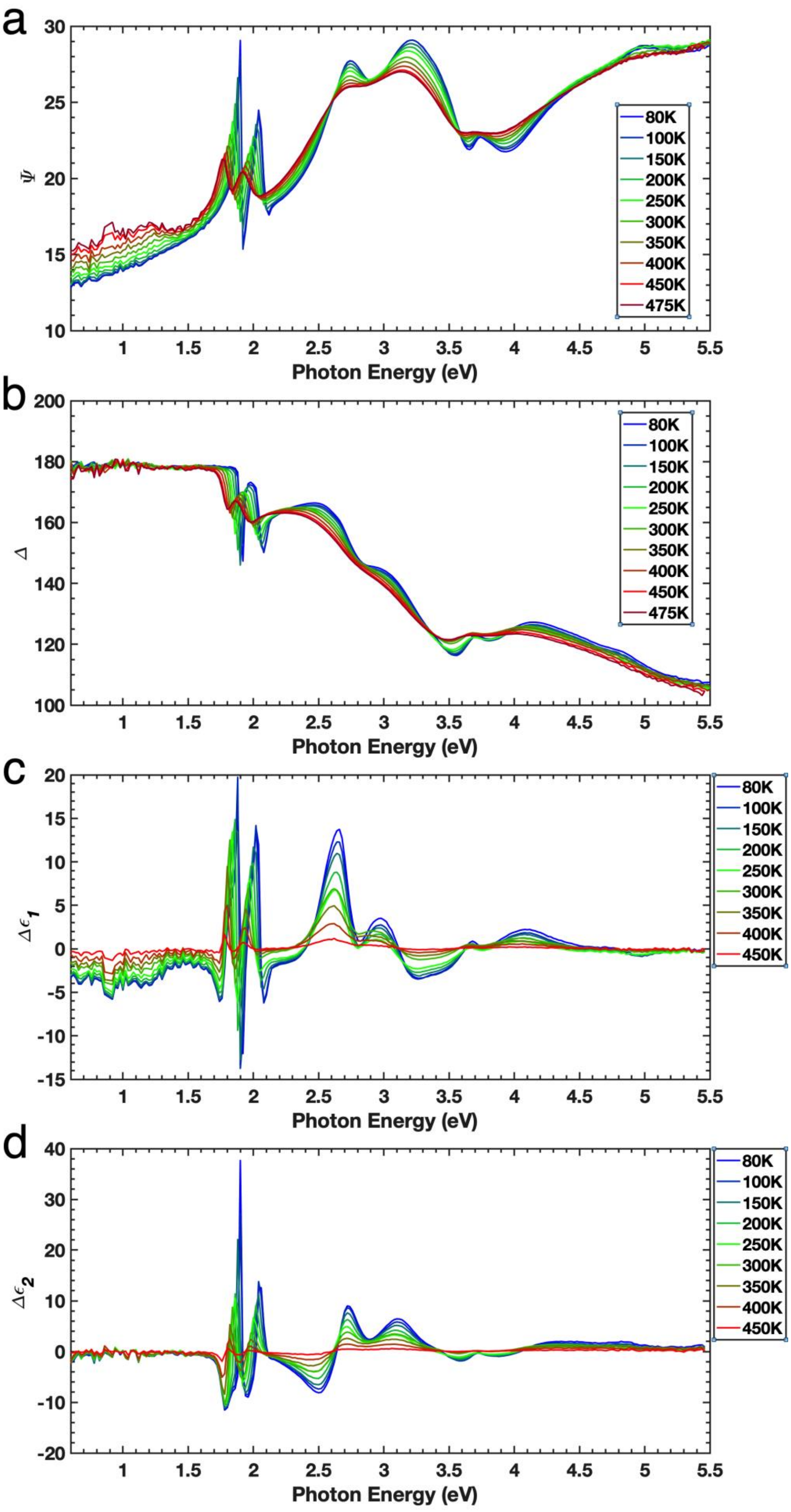

a
Ψ
Photon Energy (eV)
80K
100K
150K
200K
250K
300K
350K
400K
450K
475K
b
Δ
Photon Energy (eV)
80K
100K
150K
200K
250K
300K
350K
400K
450K
475K
c
Δϵ1
Photon Energy (eV)
80K
100K
150K
200K
250K
300K
350K
400K
450K
d
Δϵ2
Photon Energy (eV)
80K
100K
150K
200K
250K
300K
350K
400K
450K

Supplementary Figure 1 | Spectroscopic Ellipsometry measurements of MoS_2. The output data of the spectroscopic ellipsometry measurements, **a** ψ and **b** Δ, the ratio of the amplitude and the difference in phase between the incident and reflected light respectively. The difference in the **c** real and **d** imaginary parts of the dielectric function between 475 K and the colder measurements.

Muller-matrix spectroscopic ellipsometry measurements were carried out using a variable-angle spectroscopic ellipsometer (V- VASE, J.A. Woollam Co.) with a rotating analyser and compensator at the Singapore Synchrotron Light Source (SSLS). The output data from the spectroscopic ellipsometry measurements are ψ and Δ, which are shown in Supplementary Figs. 1a and 1b respectively, as a function of temperature. The measurement ψ, is the ratio of the amplitudes of the polarised light incident on the target and the polarised light reflected from the target, whilst the measurement Δ, is the phase difference between the incident and reflected polarised light.

Using the W-VASE analysis program, a model of the sample is created which includes the MoS_2 bulk material and surface effects (e.g. roughness, oxidation etc.). As the sample thickness is very large, the complex dielectric function of the MoS_2 can be determined through the best fits to the output data ψ and Δ. Single crystal MoS_2 is known to be anisotropic so the spectroscopic ellipsometer was used in the Mueller-Matrix mode with a rotational sample stage.[1,2] Supplementary Fig. 1 also shows the difference in the real, Supplementary Fig. 1c, and imaginary, Supplementary Fig. 1d, parts of the complex dielectric function between the highest temperature of 475 K measured and the lower temperatures. There is clearly a very large change in the spectral weight as the sample is cooled with the biggest changes occurring between 1.70eV and 3.40eV.

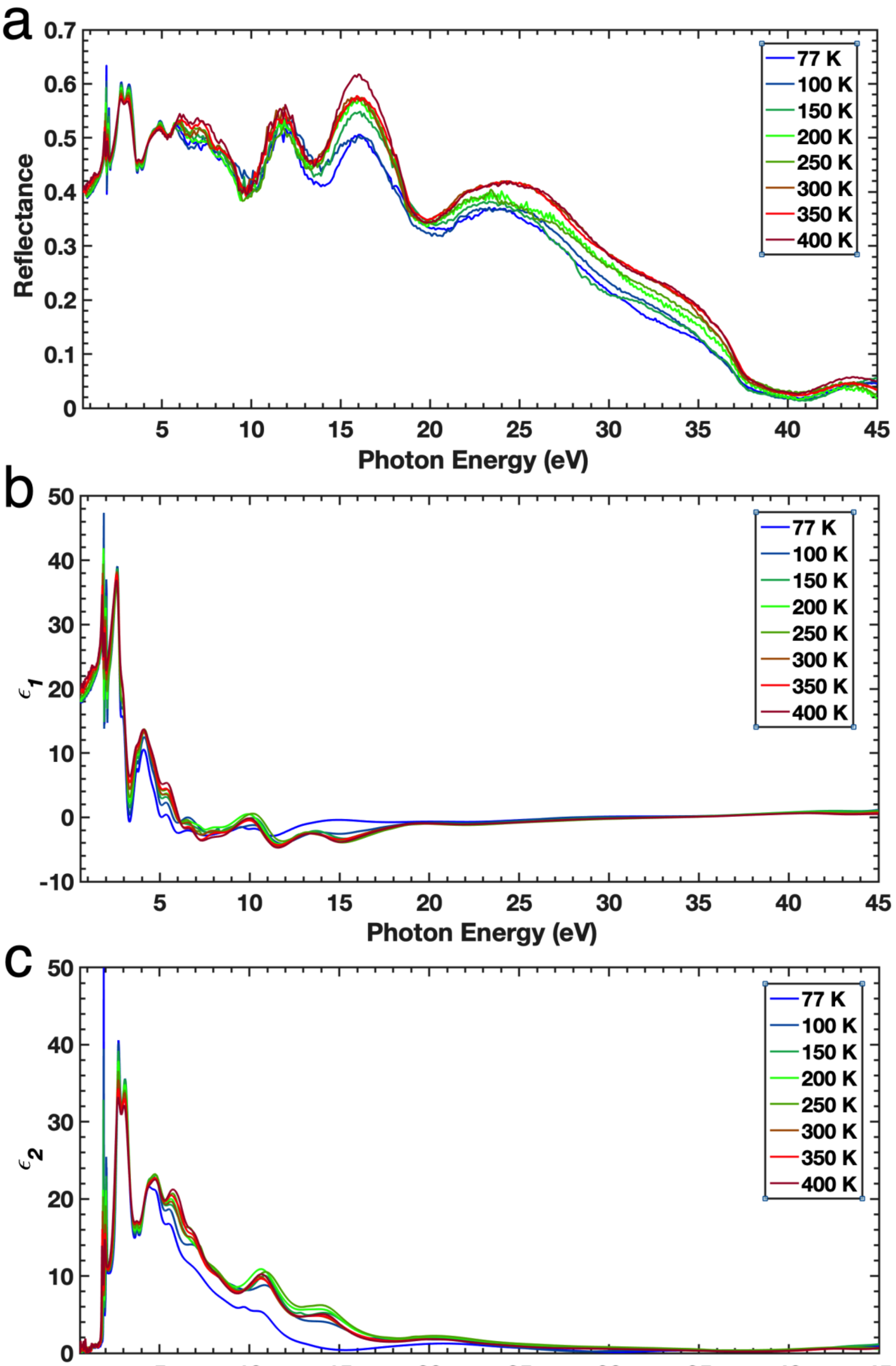

a
Reflectance
Photon Energy (eV)
77 K
100 K
150 K
200 K
250 K
300 K
350 K
400 K
b
ϵ_1
Photon Energy (eV)
77 K
100 K
150 K
200 K
250 K
300 K
350 K
400 K
c
ϵ_2
Photon Energy (eV)
77 K
100 K
150 K
200 K
250 K
300 K
350 K
400 K

Supplementary Figure 2 | Reflectance measurements and complex dielectric function of MoS_2. **a** Measurements of the reflectance of the bulk MoS_2 sample as a function of temperature from near-IR (0.6eV) up to X-ray (45eV) using the SUV beam-line at SSLS, log scale. The **b** real (ε_1) part and **c** imaginary (ε_2) part of the complex dielectric function of MoS_2 modelled from spectroscopic ellipsometry and X-ray reflectance data as a function of temperature, linear scale.

Supplementary Fig. 2a shows the combined Muller-Matrix spectroscopic ellipsometry and soft X-ray reflectivity measurements of MoS_2 as a function of temperature from 0.6eV – 45eV in the log scale. Supplementary Figs. 2b and 2c show the real, ε_1, and imaginary, ε_2, parts of the complex dielectric function, respectively, on a linear scale. The reflectivity data taken from the SUV beamline are selected in the range 3.5eV – 45eV, whilst the ellipsometry results cover the range 0.6eV – 5. 5eV, providing ~3.0eV overlap for normalization. Spectroscopic ellipsometry allows us to determine the complex dielectric function of a material from which the self-normalised reflectivity can be calculated using the following:

$$R = \frac{((n-1)^2 + k^2)}{((n+1)^2 + k^2)} \quad (S1)$$

where *n* and *k* are the refractive index and absorption coefficient respectively and can be calculated from the real (ε_1) and (ε_2) imaginary parts of the complex dielectric function using:

$$\varepsilon_1 = n^2 - k^2, \qquad \varepsilon_2 = 2nk. \ (S2)$$

The soft X-ray reflectivity is then normalised using spectroscopic ellipsometry at the lower energy range,[3] and X-ray data is tabulated at the high energy range from 30 eV onward.[4] In order to determine the complex dielectric function of the MoS_2 across the whole energy range, we use a model based on Kramers-Kronig-transformable Drude-Lorentz oscillators of the form:

$$\varepsilon(\omega) = \varepsilon_\infty + \sum_{\mathbf{k}} \frac{\omega_{p,\mathbf{k}}^2}{\omega_{0,\mathbf{k}}^2 - \omega^2 - i\Gamma_{\mathbf{k}}\omega} \quad (S3)$$

where ε_∞ is the high-frequency dielectric constant and $\omega_{p,\mathbf{k}}$, $\omega_{0,\mathbf{k}}$ and $\Gamma_{\mathbf{k}}$ are the plasma frequency, the transverse frequency (eigenfrequency), and the line width (scattering rate) of the **k**-th oscillator, respectively. Using the new methodology introduced here, our model is constrained by the complex dielectric function measured with spectroscopic ellipsometry. Therefore, we are able to obtain reflectivity with very high accuracy and resolution as a function of temperature in such a broad energy range.

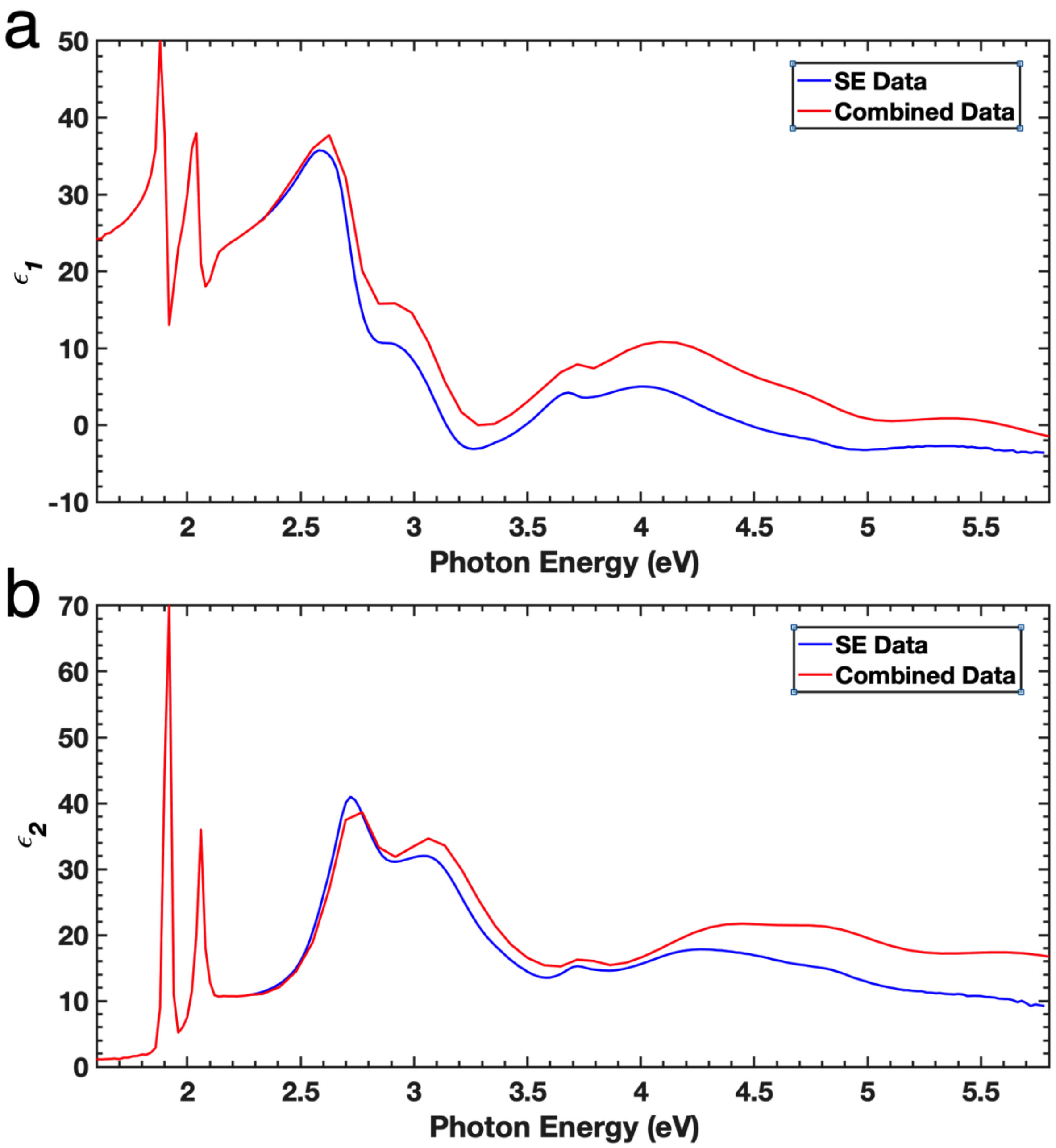

Supplementary Figure 3 | Spectral Weight Comparison of MoS_2. The **a** real and **b** imaginary parts of the complex dielectric function of MoS_2 at 40 K using just the spectroscopic ellipsometery data (blue) and from the data using a combination of spectroscopic ellipmsometry and high-energy reflectance (red).

Within a single measurement, spectroscopic ellipsometry measures the change in phase and amplitude of polarised light after being transmitted or reflected from a material,

from these we can simultaneously determine the complex dielectric function, loss function and reflectivity. For data that cover a limited spectral range, the derived complex dielectric function towards these limits is not accurate. Supplementary Fig. 3 shows the complex dielectric function determined from a small energy range (SE data) compared to a large energy range (Combined SE and high-energy reflectivity data). It is clear that the complex dielectric function is severely underestimated above 2.5eV using data from only a limited energy range. Therefore, low energy measurements, especially those trying to determine the complex dielectric function and spectral weight transfer, are not always valid. Higher energy band measurements must also be taken into consideration.

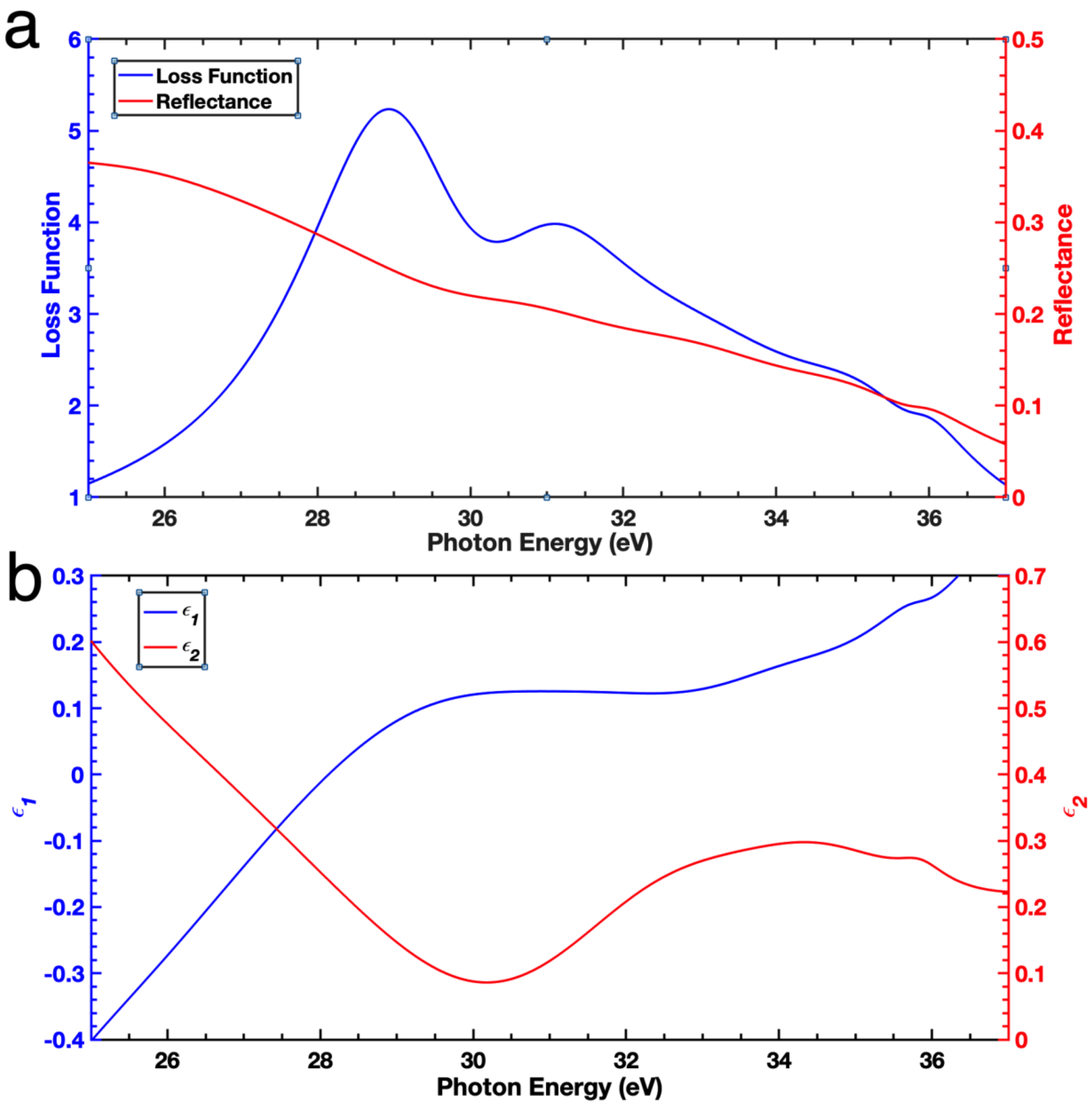

Supplementary Figure 4 | Loss function, complex dielectric function and reflectivity of 40K MoS_2. A comparison of **a** the loss function and reflectivity, and **b** the complex dielectric function of 40 K MoS_2 in the energy range 25 – 37eV in order to show the properties of soft X-ray correlated-plasmons.

Supplementary Fig. 4 shows the loss function, reflectivity, and complex dielectric function of MoS_2 cooled to 40K. Correlated plasmons have the following characteristics; (1) peaks in loss function as seen at 30eV in Supplementary Fig 4a, (2) local minimums in

reflectivity, and (3) near zero values of ε_1 as seen at the same energy in Supplementary Fig 4b. What makes it different from metal is the value of ε_1. Unlike conventional plasmons in metal that have negative values of ε_1, the ε_1 in correlated-plasmons have values that are near zero due to electronic correlations.

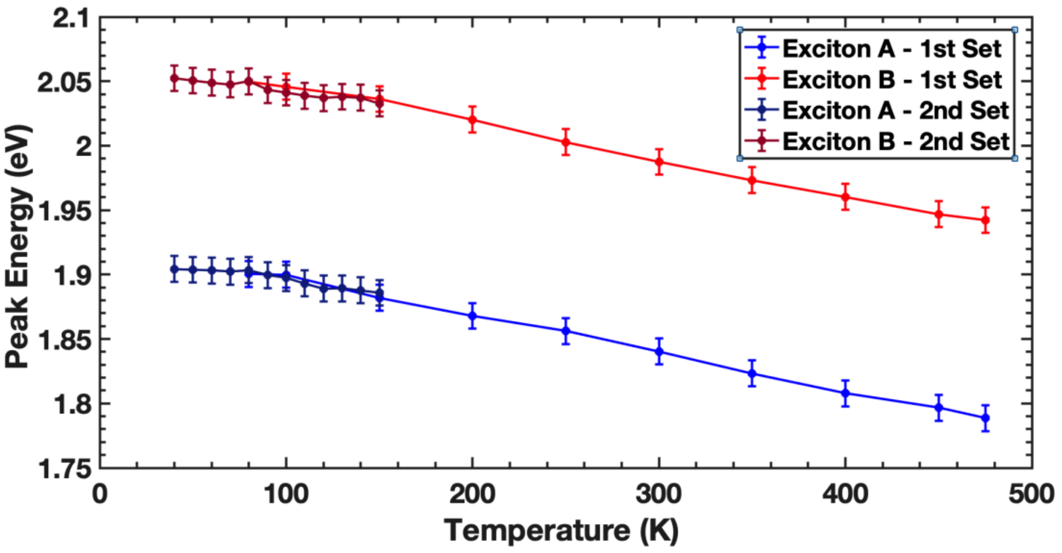

Supplementary Figure 5 | Peak position of excitons A and B. The peak positions of excitons A and B as a function of temperature from 475K to 40K for both sets of measurements. Error bars are calculated using a 2meV shift.

Supplementary Fig. 5 shows the peak positions of excitons A and B measured from the spectroscopic ellipsometry results in Fig. 1c of the main text. These include measurements from the first set of results seen in Figs. 1a-c and the second set of results with temperatures measured from 150K down to 40K at 10K intervals, both of which are in excellent agreement with each other. There is a steady blue-shift of the peak position as the temperature is decreased from 475K to 40K.

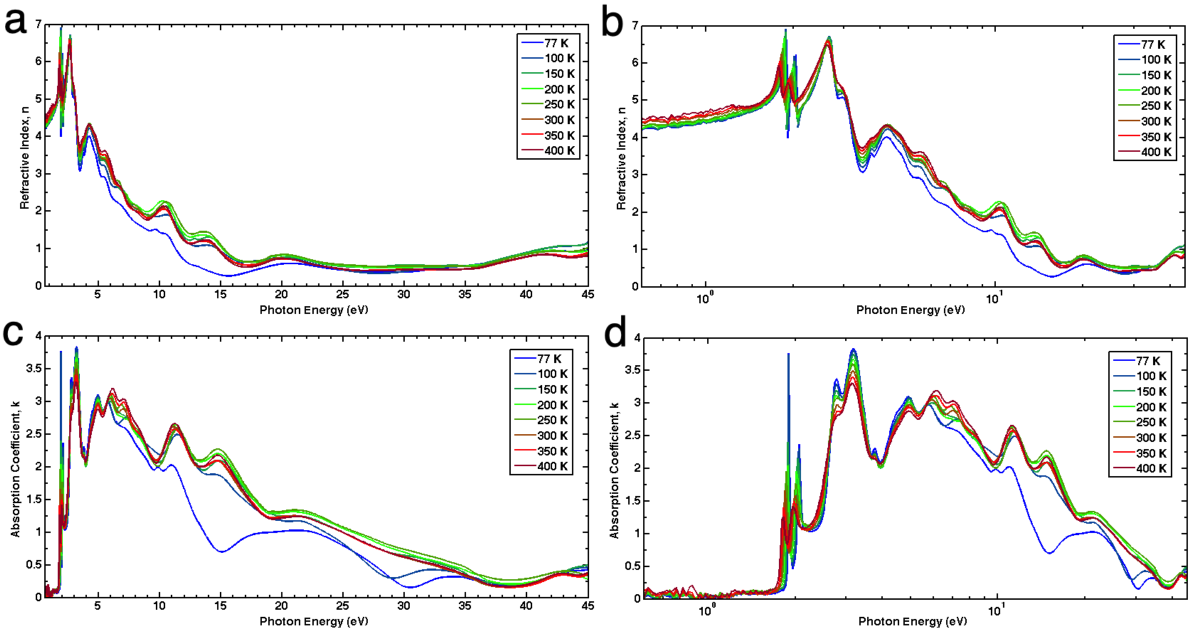

Supplementary Figure 6 | Complex refractive index of MoS_2. The refractive index, *n*, of MoS_2 as a function of temperature **a** linear scale, **b** log scale. The absorption coefficient, *k*, of MoS_2 as a function of temperature **c** linear scale, **d** log scale.

Supplementary Fig. 6 shows the complex refractive index of MoS_2 as a function of temperature from 0.6eV – 45eV. Supplementary Figs. 6a and 6b show the linear and log plots of the refractive index, *n*, respectively and Supplementary Figs. 6c and 6d show the linear and log plots of the absorption coefficient, *k*.

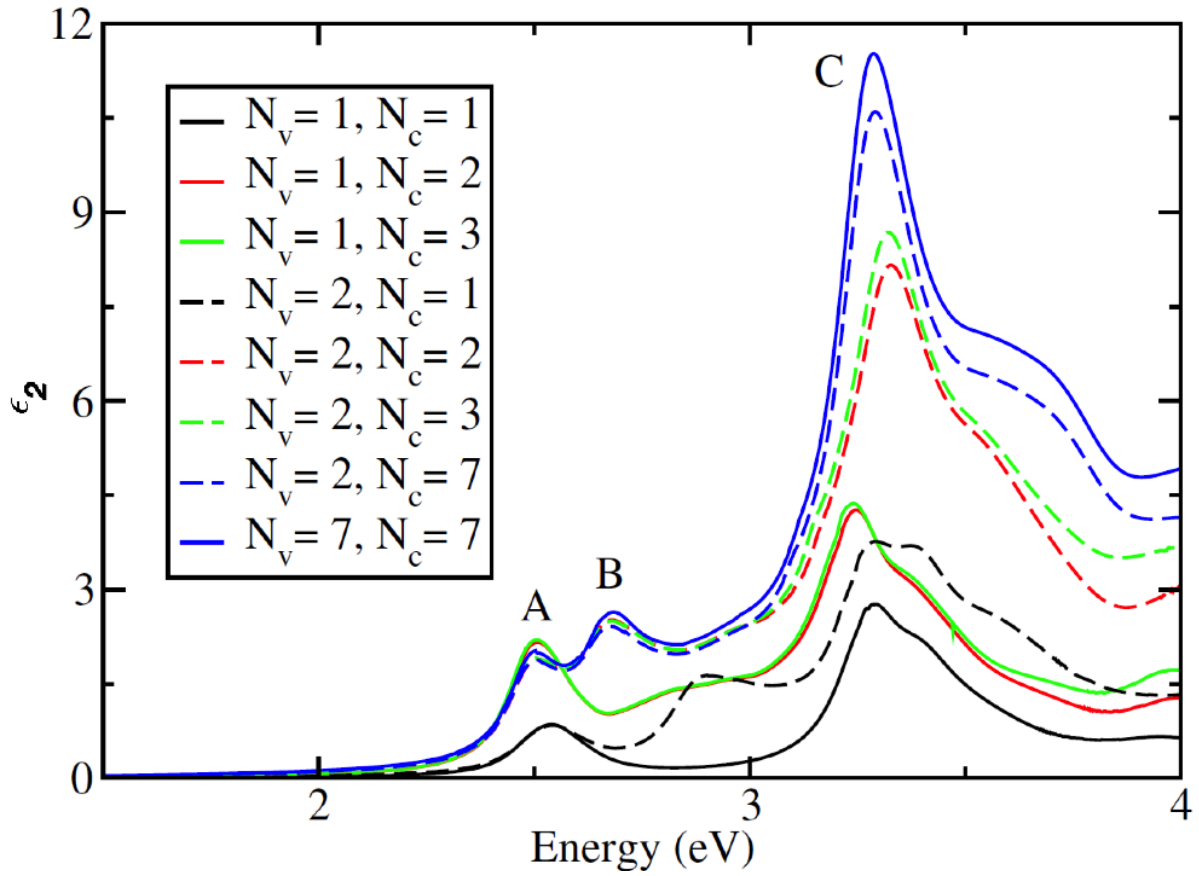

Supplementary Figure 7 | Calculated ε_2 as a function of the number of bands. Theoretical calculations based on GW+BSE for different number of valence bands N_v and conduction bands N_c.

We perform theoretical calculations by varying the number of valence bands (N_v = 1, 2 and 7) and conduction bands (N_c = 1, 2, 3 and 7) of MoS_2 and calculate ε_2 by incorporating electron-electron and electron-hole interactions using *GW* Bethe-Salpeter equation (BSE) calculations.[5] More numbers of valence and conduction bands mean that there are more higher energy bands involved and a higher number of valence and conduction bands is needed to properly calculate the spectrum in the energy range of interest. We utilize $Nv = Nb = 7$ to give a converging result.

From Supplementary Fig. 7, we see that the single-band transition $N_v = N_c = 1$ can already form the excitonic peaks A and C but the excitonic peak B is missing. By increasing the number of conduction bands at high energies, we can see that low-energy excitations are enhanced, including these excitons. This further supports that the low-energy excitations are determined by high-energy bands. We note that our calculated values for the three excitonic peaks are in average overestimated by ~0.57 eV, which is due to the limited of number of *k*-points in the GW calculations.

Supplementary References:

1. Fujiwara, H., *Spectroscopic ellipsometry: principles and applications*. (John Wiley & Sons, 2007).
2. Schmidt, D., You, L., Chi, X., Wang, J., & Rusydi, A., Anisotropic optical properties of rhombohedral and tetragonal thin film $BiFeO_3$ phases, *Phys. Rev. B* **92**, 075310 (2015).
3. Whitcher, T. J., *et al.*, Correlated plasmons in the topological insulator Bi_2Se_3 induced by long-range electron correlations, *NPG Asia Mater.* **12**, 1 (2020).
4. Henke, B.L., Gullikson, E.M., & Davis, J.C., *X-ray interactions: photoabsorption, scattering, transmission, and reflection at E=50-30000 eV, Z=1-92,* Atomic Data and Nuclear Data Tables, **54**, 181 (1993).
5. Onida, G., Reining, L. and Rubio, A., Electronic excitations: density-functional versus many-body Green's-function approaches. *Rev. Mod. Phys.* **74**, 601 (2002).
